# A large-scale phylogeny-guided analysis of pseudogenes in *Pseudomonas aeruginosa* bacterium

**DOI:** 10.1128/spectrum.01704-23

**Published:** 2023-09-26

**Authors:** Nimrod Cohen, Isana Veksler-Lublinsky

**Affiliations:** 1 Department of Software and Information Systems Engineering, Faculty of Engineering, Ben-Gurion University of the Negev, Beer-Sheva, Israel; University at Albany, Albany, New York, USA

**Keywords:** pseudogenes, phylogenetics, bacteria, *Pseudomonas aeruginosa*, comparative genomics

## Abstract

Pseudogenes, once considered "junk DNA" based on the incorrect assumption that the absence of full coding potential means a complete lack of functionality, have recently become a subject of significant interest in the scientific community. Concurrently, it is widely assumed that bacterial genomes are compact and have a high density of coding genes with little room for non-coding genes, including pseudogenes. A key aspect of genome annotation is the correct identification of genes and the distinction between coding genes and pseudogenes, as it directly impacts functional and comparative genomics studies. In this study, we analyzed the genomic data of 4,699 strains of the bacterium *Pseudomonas aeruginosa* (*P. aeruginosa*) as they exhibit high variability in the number of annotated pseudogenes. In particular, we looked for correlations between the number of pseudogenes and other genomic and meta-features of the strains. We identified clusters of orthologous genes and pseudogenes and compared cluster size distributions and length homogeneity within clusters. We then mapped and examined orthology relationships between genes and pseudogenes. Additionally, we generated a phylogenetic tree of the strains and found that phylogenetically related strains are more homogeneous in the number of pseudogenes and share a significant amount of pseudogenes. Finally, we delved into clusters of orthologous genes and pseudogenes and quantified their phylogenetic neighborhood, classifying pseudogenes into evolutionary preserved pseudogenes, mis-annotated pseudogenes, or pseudogenes formed by failed horizontal transfer events. This in-depth study provides important insights that can be incorporated into pseudogene annotation pipelines in the future.

**IMPORTANCE:**

Accurate annotation of genes and pseudogenes is vital for comparative genomics analysis. Recent studies have shown that bacterial pseudogenes have an important role in regulatory processes and can provide insight into the evolutionary history of homologous genes or the genome as a whole. Due to pseudogenes’ nature as non-functional genes, there is no commonly accepted definition of a pseudogene, which poses difficulties in verifying the annotation through experimental methods and resolving discrepancies among different annotation techniques. Our study introduces an in-depth analysis of annotated genes and pseudogenes and insights that can be incorporated into improved pseudogene annotation pipelines in the future.

## INTRODUCTION

Pseudogenes are gene fragments that have undergone partial or full disablement of the original gene function (1). It is largely assumed that bacterial genomes are compact and efficient and have a high density of coding genes with little room for non-coding genes, including pseudogenes (2). Bacterial pseudogenisation occurs in various ways, including horizontal gene transfers that result in the silencing of most genes, adopting to an intracellular lifestyle within a host which can lead to destabilized genome, and continuous absence of selective pressure that can lead to accumulation of mutations (3, 4). Thus, pseudogenes can serve as a record of the proteins or pathways that were no longer required when adapting to a new environment or host (3).

The dramatic decrease in the time and cost of high-throughput sequencing in recent years enabled the sequencing and assembly of thousands of new genomes; still, annotation errors remain prevalent as in the past (5, 6). A key aspect of correct annotation is identifying genes and accurately distinguishing between coding genes and pseudogenes. Sequencing errors, incomplete genome assembly, and varying annotation approaches across databases can lead to the erroneous annotation of a functional gene as a pseudogene (7) or vice versa (8), affecting their inclusion in comparative genomics analysis. Furthermore, the absence of a widely agreed-upon pseudogene definition hinders experimental validation and agreements among the annotation methods (9).

Some attempts have aimed to address these challenges. The tool *PseudoPipe* (10) identifies pseudogenes by searching for all regions in the genome that share sequence similarity with known proteins and then applies post-processing and filtering steps. *Pseudofinder* (11) is a tool designed for identifying pseudogenes in bacterial genomes, and it incorporates evolutionary metrics such as *dN*/*dS* (12) from closely related genomes, in addition to sequence homology. *SearchDOGS Bacteria* (8) is a comparative genomics and synteny-based tool designed to identify potential missing genes, including those annotated as pseudogenes, in bacterial genomes. Nevertheless, accurate identification of pseudogenes remains a complicated task, partly due to the difficulty distinguishing between genuine frameshift mutations [that can lead to pseudogenisation, but can be well preserved for functional purposes as reported in references (13 – 15)] from sequencing errors, and thus must be supplemented by manual inspection and curation.

Several studies analyzed and characterized pseudogene populations in bacterial genomes. Liu et al. (16) surveyed 64 prokaryote genomes, identified that at least 1%–5% of all gene-like sequences are pseudogenes, and concluded that the estimation of actual pseudogene numbers could vary substantially if different criteria for pseudogene identification are used. In their review, Goodhead and Darby (3) mentioned several studies where manual annotation identified additional pseudogenes that automatic pipelines failed to identify. A recent study in *Sodalis glossinidius*, which contains a relatively high portion of pseudogenes (4), reported that some pseudogenes are expressed and take an active role in control mechanisms, posing another difficulty in pseudogene identification.

In this study, we aimed to characterize the phylogenetic patterns of annotated pseudogenes in the highly studied bacterium *Pseudomonas aeruginosa* (*P. aeruginosa*), as there are thousands of sequenced and annotated genomes showing high variability in the number of pseudogenes. To that end, we collected genomic data of *P. aeruginosa* strains and examined correlations between the number of pseudogenes with other factors. Then, we clustered genes and pseudogenes separately and found differences in cluster size distributions and length homogeneity of the clustered sequences. Using an additional clustering round, we mapped and examined orthology relationships between genes and pseudogenes. Using this methodology, we identified strains that are enriched with pseudogenes without orthologous pseudogenes, which could point to poorly annotated strains. In addition, we generated a phylogenetic tree of all strains and grouped strains by different criteria. We found that phylogenetically related strains are more homogeneous in the number of pseudogenes and some share a significant amount of pseudogenes.

Finally, we delved into clusters of orthologous genes and pseudogenes and quantified the phylogenetic neighborhood of each sequence. We propose that the results of such analysis may help classify pseudogenes into evolutionary preserved pseudogenes, mis-annotated pseudogenes, or pseudogenes formed by failed horizontal transfer events. We found that many pseudogene sequences fall within the second category. While most of the strains in our analysis are of low assembly quality, which mirrors the state in other bacteria as well, this abundance of data allowed us to propose a phylogeny-based approach that captures the weaknesses (i.e., mis-annotated pseudogenes) within individual strains. This in-depth study of the orthology-based features of the annotated pseudogenes provides important insights that can be incorporated into pseudogene annotation pipelines in the future.

## RESULTS

### Genomic characteristics of *P. aeruginosa* strains

We collected the genomic data of 4,699 strains of *P. aeruginosa* (Table S1) and analyzed various genomic characteristics, e.g., genome size and number of genes and pseudogenes. The genome size of the strains in our collection ranges from 5.3 to 7.8 Mb with an average size of 6.64 Mb (Fig. S1a). The number of genes ranges from 4,844 to 7,507 with an average of 6,160 genes (Fig. S1b), while the number of pseudogenes ranges from 0 to 1,970 with an average of 237 pseudogenes per strain (Fig. S1c). The distributions of the genome size and the number of genes are symmetric and bi-modal, while the distribution of the number of pseudogenes is skewed to the left with a long tail to the right.

We next examined correlations between the variables. Our hypothesis is that in the case of misannotation of genes as pseudogenes, we would likely see a lower number of genes and a higher number of pseudogenes. Thus we expected to see an inverse correlation between the number of pseudogenes and the number of genes. We found that there is a correlation between genome size and the number of genes (Pearson’s *r* = 0.349, *P* value = 2.7e*−*135, Fig. S1d). However, we found there is no correlation between the number of pseudogenes to the number of genes (Pearson’s *r* = 0.0151, Fig. S1e), nor the genome size (Pearson’s *r* = −0.0108, Fig. S1f). Nevertheless, several strains have a high number of pseudogenes and a low number of genes.

**Fig 1:**
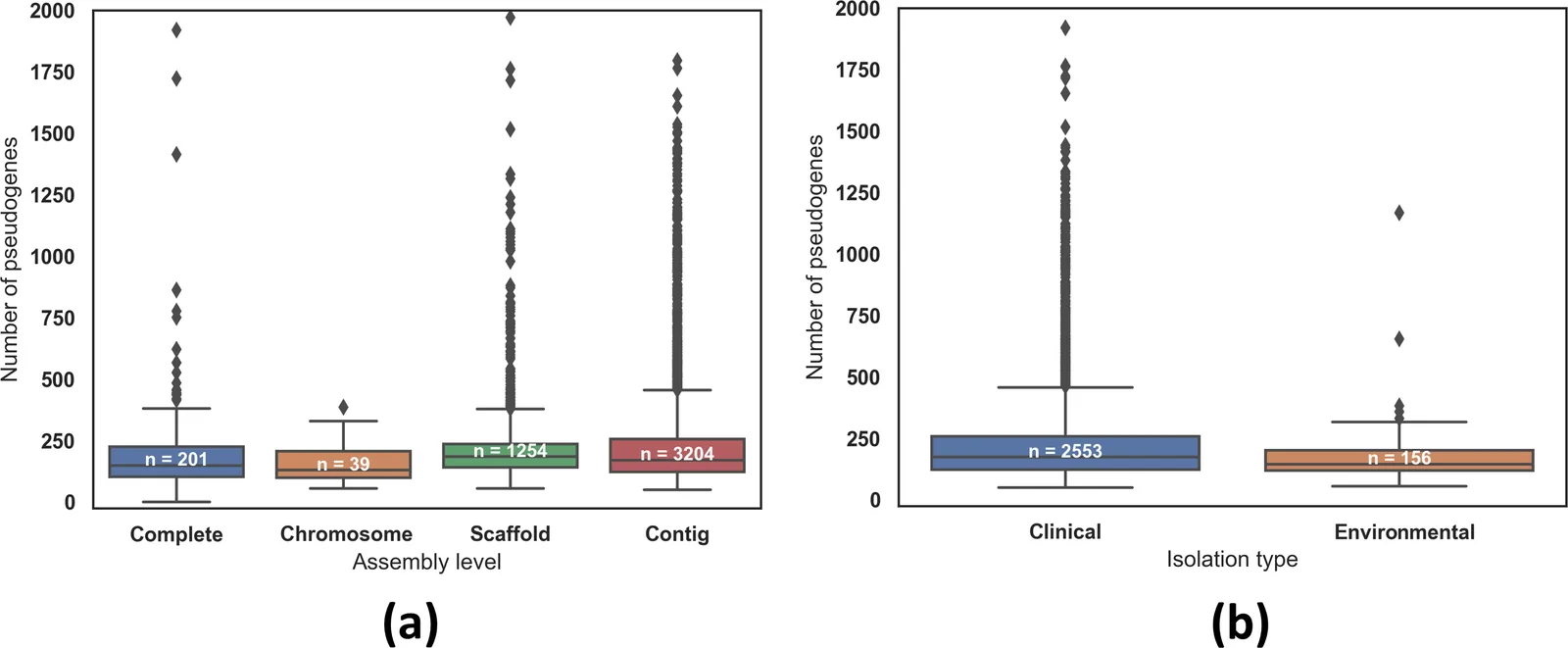
Association between the number of pseudogenes and (a) assembly level and (b) isolation type of the strains. For each group, the number of observations denoted by *n* is shown above the median line. The box and whiskers span the interquartile range (IQR) and 1.5 × IQR, respectively, and diamonds represent outliers. One strain and 1,990 strains are missing information about assembly level and isolation type, respectively, and thus were dropped from the plots.

As the number of pseudogenes varied widely between strains, we examined if there is an association between the genome-assembly level of the strains (*complete*, *chromosome*, *scaffold*, or *contig*) and their number of pseudogenes (Fig. 1a). Most strains are assembled to low levels, i.e., scaffold and contig. We hypothesized that lower assembly levels are more prone to sequencing or annotation errors which may lead to the misannotation of genes as pseudogenes and, thus a higher number of pseudogenes compared to higher assembly levels. We found that there are statistical differences between the complete/chromosome and contig/scaffold groups (Dunn’s test, *P* value < 0.05), as evident also by higher medians for the latter two.

Next, we examined the isolation type of the strains (clinical or environmental) as an additional source of variation that may associate with the number of annotated pseudogenes in their genomes (Table S1). The majority of the strains were acquired from clinical samples (Fig. 1b), yet showed a statistically higher number of pseudogenes than the environmental strains (Wilcoxon-Mann-Whitney test, *P* value = 4.46e*−*05).

### Clustering of coding genes and pseudogenes reveals substantial differences in the cluster size frequency and length homogeneity

**Fig 2:**
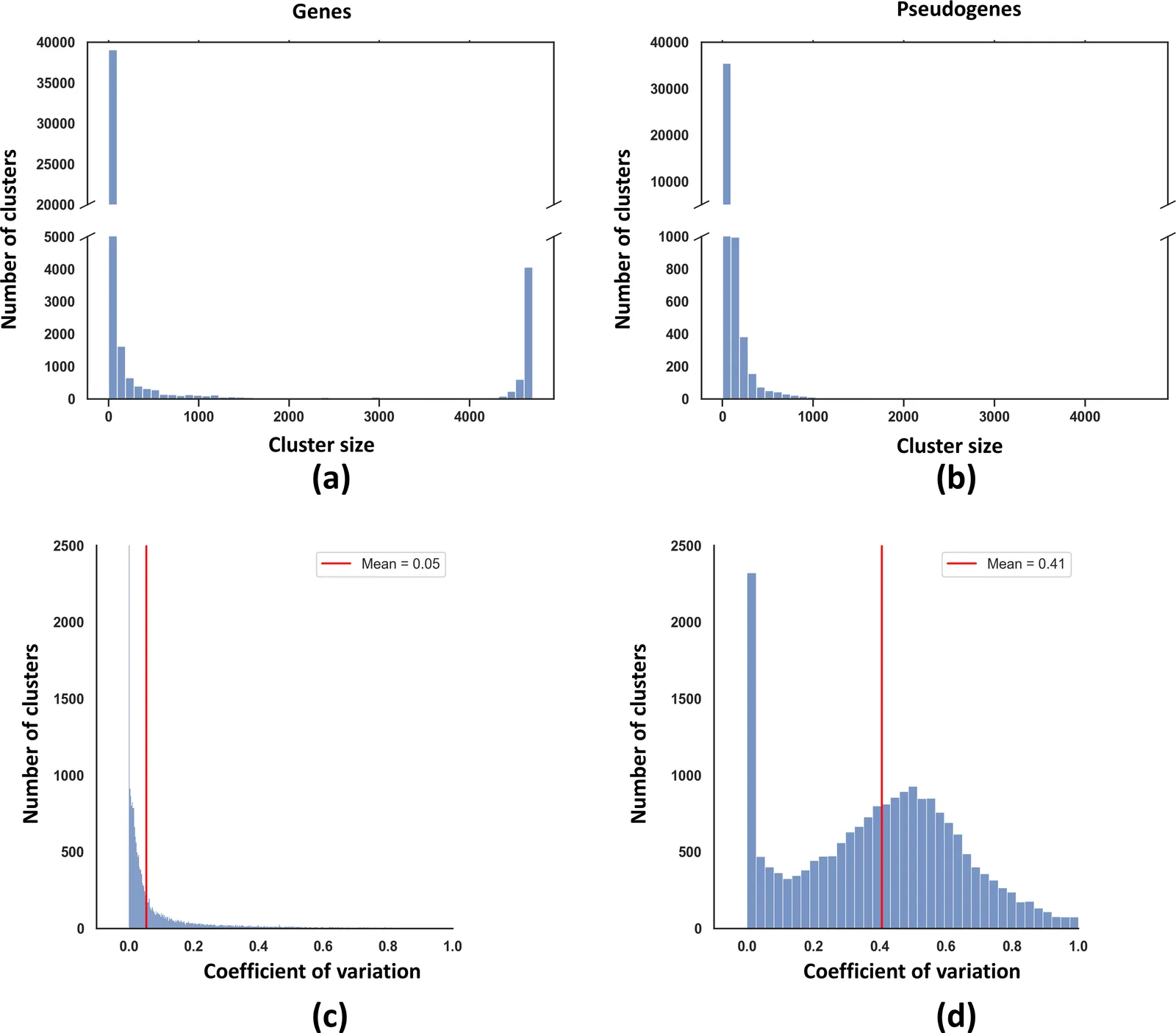
Distributions of cluster size and sequence length variability within gene and pseudogene clusters. (a and b) Distributions of cluster size in terms of the number of strains represented in the cluster of (a) gene and (b) pseudogene. In both graphs, the leftmost peak represents "singletons" (clusters that consist of genes/pseudogenes that appear in only one strain) and rare accessory genes/pseudogenes. In (a), the rightmost peak represents core genes that are present in a majority of the strains. (c and d) Distribution of coefficient of variation (CV) of sequence length within (c) gene and (d) pseudogene clusters. For a better view, the graph in (c) was cut in the *y*-axis (max = 16,322), and both (c) and (d) were cut in the *x*-axis (max = 1.9).

To investigate the phylogenetic distribution of homologous pseudogenes and to identify their connections to genes, we performed several rounds of clustering based on sequence similarity. First, we clustered the protein-coding genes (altogether 28,948,105 sequences) of all strains using CD-HIT (17) with a threshold of 70% amino-acid similarity; 48,968 distinct gene clusters (i.e., groups of homologous genes) were identified (Table S2). We then examined the distribution and frequency of these gene clusters across all strains (Fig. 2a). The rightmost peak represents the 5,011 core genes present in more than 90% of the strains; 4,651 were present in the strains in a single copy and were utilized for phylogenetic analysis (see below). The remaining 43,957 gene clusters represent accessory genes, associated with more unique functions, such as adaption to a new environment or host and antibiotic resistance. A total of 13,021 clusters are "singletons", consisting of rare genes that appear only in one strain. A bimodal distribution was previously also reported for the pangenome analysis of a smaller set of 1,488 *P*. *aeruginosa* strains (18).

Next, we studied the distribution of pseudogenes in the same manner as for coding genes. A total of 1,113,358 pseudogene sequences were clustered with CD-HIT-EST, using DNA sequences (since no proteins can be translated from pseudogenes) and a threshold of 80% nucleotide similarity. A total of 37,305 distinct pseudogene clusters were identified (Table S2), representing between 1 and 4,679 strains, yet 90% of the clusters contain pseudogenes from less than 50 strains. A total of 11 clusters are present in more than 90% of the strains (i.e., core clusters). A total of 17,555 clusters are singletons, indicating these pseudogenes have no homologous pseudogenes in other strains (Fig. 2b). The majority of pseudogene clusters are sparser (i.e., represent sequences from a smaller number of strains) compared to gene clusters. While both histograms show similar frequency distribution, the pseudogenes distribution is missing the right peak representing the core clusters group.

We hypothesized that gene clusters are more homogeneous in length than pseudogene clusters. To test that, we calculated the coefficient of variation (CV) of the sequences’ length within each gene/pseudogene cluster. Singleton clusters containing sequences from only one strain were omitted from the analysis (thus, 35,947 and 19,751 gene and pseudogene clusters, respectively, were analyzed), and each gene sequence length was multiplied by 3 (to make them comparable to pseudogenes). The distribution of gene cluster CV is skewed to the left with an average of 0.05 (75th percentile = 0.04, max = 1.9) (Fig. 2c), while the pseudogene clusters’ CV is normally distributed (excluding the left tail) with an average of 0.41 (75th percentile = 0.58, max = 1.8) (Fig. 2d). In addition, 16,322 gene clusters (45%) compared to 1,508 pseudogene clusters (7%) had a CV = 0. These results confirm that pseudogene clusters have much higher sequence length variability than gene clusters.

### Clustering of representative pseudogenes and genes maps their orthology relationships

To understand the orthology relationships between genes and pseudogenes, we aggregated the DNA sequences of the representatives from the gene and the pseudogene clusters (48,968 and 37,305, respectively) and then clustered them together (80% similarity). We identified 67,349 clusters (Table S2), containing various combinations of representatives: 33,420 are gene-only, 20,419 are pseudogene-only, and 13,510 are mixed clusters of both genes and pseudogenes (Table S3).

**Fig 3:**
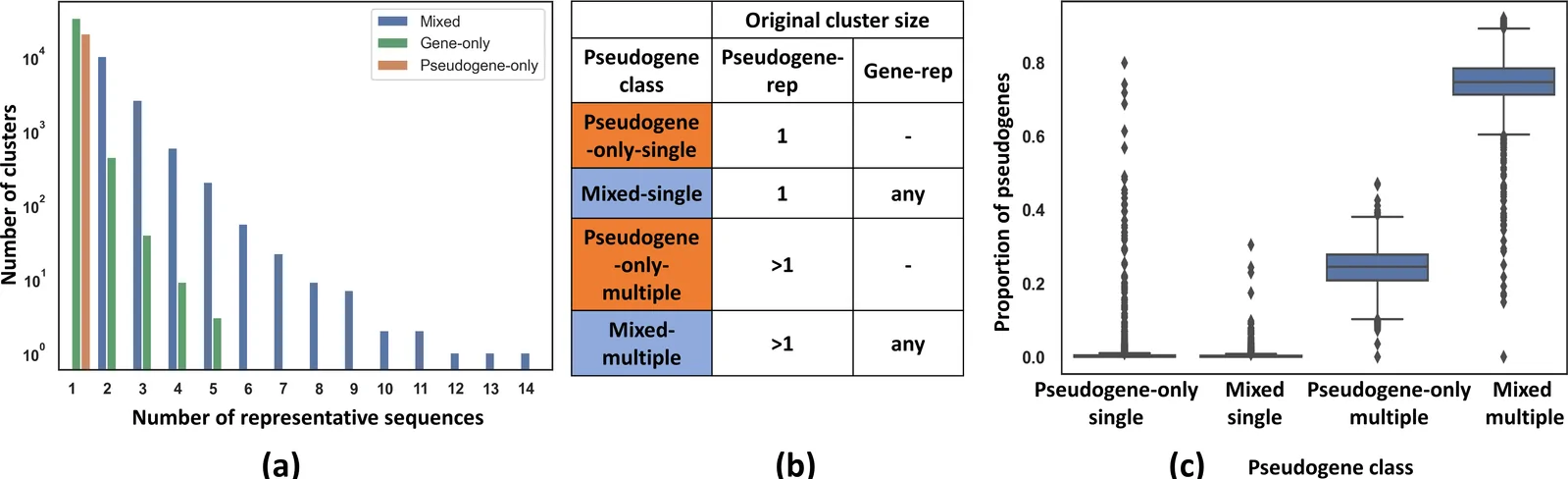
(a) Distribution of the number of sequences in gene/pseudogene representative clusters. The plot is in log scale. Clusters are labeled based on the content of the clusters gene-only*,* pseudogene-only, or mixed. (b) Further classification of pseudogene-only and mixed clusters, based on the size of the original clusters. (c) Distribution of pseudogenes by cluster classification. Each strain’s pseudogenes are classified into one of the classes shown in (b). The proportions of each class across all strains are plotted. The box and whiskers span IQR and 1.5 × IQR, respectively, and diamonds represent outliers. Distribution parameters matching the box plots are found in Table S4.

We examined the distribution of the number of representative sequences in the clusters and their origin (i.e., gene or pseudogene cluster) (Fig. 3a; Table S3). Most of the clusters (79.2%) contain one representative sequence; gene-only (32,931, 48.9%) or pseudogene-only (20,419, 30.3%). The next largest group represents clusters with two representatives, one from a gene cluster and one from a pseudogene cluster denoted as mixed (10,036, 14.9%). The remaining clusters are mixed with more than two representatives (5.2%) or gene-only with two or more representatives (0.7%). We extracted the sizes of the clusters from which the representative sequences originated to examine if there is a bias in cluster size in gene-only, pseudo-gene-only, or mixed clusters. As previously, cluster size corresponds to the number of unique strains represented by the sequences in the cluster. Interestingly, gene-only clusters (Fig. S2a), pseudogene-only clusters (Fig. S2b), as well as mixed clusters (Fig. S2c) showed similar distributions to the total clusters population (Fig. 2); all distributions are skewed to the left, where the gene clusters’ distributions both for gene-only and for mixed clusters also contain a right peak that represents the core clusters, indicating there is no bias in the original gene and pseudogene clusters toward a specific representative-cluster type.

To measure the extent to which pseudogene sequences have orthologous pseudogenes and/or genes and to examine if there are biases in specific strains, we focused on pseudogene-only and mixed clusters. We further divided the clusters based on the size of the original clusters as follows: (1) pseudogene-only-single: pseudogene has no ortholog pseudogenes or genes; (2) mixed-single: pseudogene has no ortholog pseudogenes but has ortholog genes; (3) pseudogene-only-multiple: the pseudogene has ortholog pseudogenes but no ortholog genes; and (4) mixed-multiple: the pseudogene has ortholog pseudogenes and genes (Fig. 3b).

Then, for each strain, we calculated the proportion of pseudogenes in each of the classes (Table S1) and plotted the distributions of the proportions across all strains (Fig. 3c). For most strains, the majority of pseudogenes belong to the fourth class (i.e., have orthologous pseudogenes and genes) with a mean proportion of 0.748. High proportions of pseudogenes fall in the third class (i.e., have orthologous pseudogenes) with a mean proportion of 0.241, while the first two classes have a mean proportion of 0.03 and 0.08. Nevertheless, for some strains, the proportions of pseudogenes with no orthologous pseudogenes (classes 1 and 2) are high and reach 0.8 and 0.304, respectively; for 42 strains, the proportion of pseudogenes in one of these two classes is above 0.2. These observations suggest that single pseudogenes (with no orthologs in other strains) belong to a few strains, which could further imply that these strains are poorly annotated.

### Phylogenetic analysis of pseudogenes confirms that phylogenetically related strains are more homogeneous in the number of pseudogenes

As the number of pseudogenes across strains varied widely, we assessed whether phylogenetically related strains show higher homogeneity in the number of pseudogenes compared to random groups. To that end, we generated a phylogenetic tree of *P. aeruginosa* strains and defined several groupings of the strains, i.e., by (1) adjacency on the phylogenetic tree; (2) multilocus sequence typing (MLST); (3) BioProject; and (4) random.

**Fig 4:**
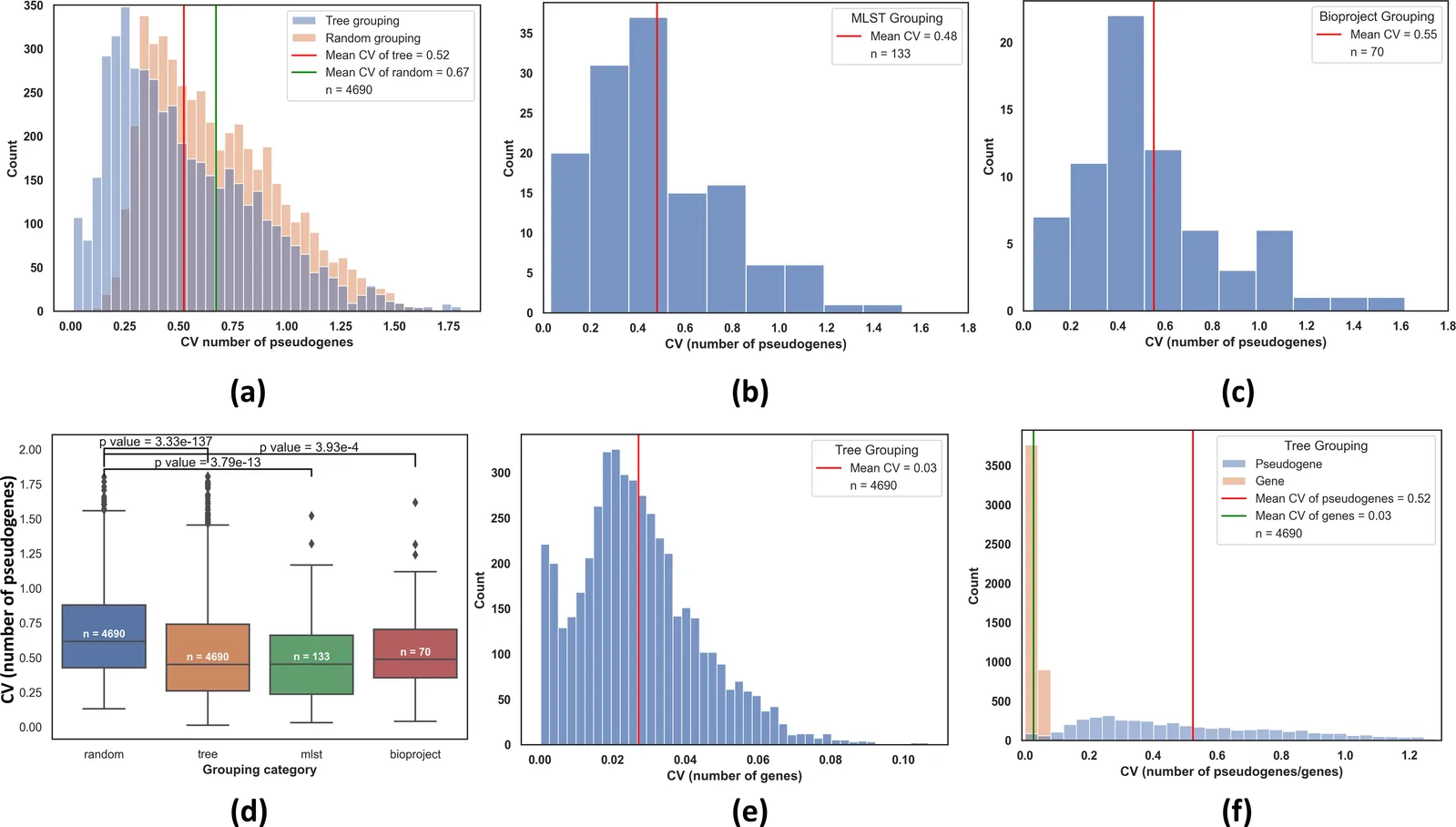
Distribution of CV of the number of pseudogenes or genes for different groupings of strains. (a–d) CV of the number of pseudogenes. (a) Grouping by phylogenetic tree versus random grouping. (b) Grouping by MLST. (c) Grouping by BioProject. (d) Comparison of CV distribution between different groupings (*P* values are computed with Mann-Whitney *U* rank test, shown are only significant *P* values <0.05). The box and whiskers span IQR and 1.5 × IQR, respectively, and diamonds represent outliers. (e) CV of the number of genes; grouping by phylogenetic tree. (f) CV of the number of genes versus CV of the number of pseudogenes; grouping by phylogenetic tree. Group size in (a) and (d–f) is 9. A similar analysis for group size 15 can be found in Fig. S3.

MLST is a scheme for identifying relationships among bacteria by examining multiple, usually seven, housekeeping gene loci, which was shown to infer evolutionary closeness in bacteria (19, 20). A BioProject is a collection of biological data related to a single initiative from a single organization or a consortium. However, there is no guarantee that the sequenced strains are phylogenetically close. There are 133 MLST groups (containing between 5 and 245 strains) and 70 BioProject groups (containing between 5 and 846 strains). The median number of strains in MLST and BioProject groups is 9 and 15, respectively. Thus, we used a sliding window of sizes 9 and 15 to traverse the tree and retrieve 4,690 and 4,684 groups, respectively, of adjacent strains. Finally, we also sampled 4,690 and 4,684 random groups of size 9 and 15, respectively, ignoring any known relationships between the strains. For each group of strains in all categories, we computed the CV of the number of pseudogenes (Fig. 4; Fig. S3). We observed similar trends for group sizes 9 and 15. Thus, we focus on size 9. The values of CV in the tree grouping range from 0.01 to 1.8 (mean = 0.52), while for random grouping, they range from 0.13 to 1.8 (mean = 0.67) with a shift toward larger values (Fig. 4a). The mean CV of the MLST grouping distribution is equal to 0.48 (Fig. 4b), while the mean of BioProject distribution is higher and equals to 0.55 (Fig. 4c).

Based on the nonparametric Mann-Whitney *U* rank test, the CV values of random groups are significantly larger than the CV values of other groups, with *P* values 3.33e−137, 3.79e−13, and 3.93e−4 for the tree, MLST, and BioProject groupings, respectively (Fig. 4d), supporting the hypothesis that the number of pseudogenes within phylogenetically related strains is less variable than within strains picked randomly.

For comparison, we also computed the CV of the number of genes in the tree grouping for window sizes of 9 and 15. The CV values are much smaller compared to pseudogenes, ranging in 0.0002–0.107 and 0.0002–0.084 for window sizes of 9 and 15, respectively (Fig. 4e and f; Fig. S3c and d), with a mean CV of 0.03 in both window sizes. This comparison confirms that the number of genes across groups of phylogenetically related strains is less variable than the number of pseudogenes.

We visualized the distributions of the number of genes and the number of pseudogenes on top of the phylogenetic tree, coloring the branches of the tree based on MLST (Fig. S4) or BioProject (Fig. S5). Examination of these trees confirms that the total number of genes is less variable than the number of pseudogenes, in agreement with the above analysis. In addition, it is evident that strains that are close in some areas of the tree often share the same MLST and demonstrate similar pseudogene counts.

### Some strains that are phylogenetically close share a significant amount of pseudogenes

**Fig 5:**
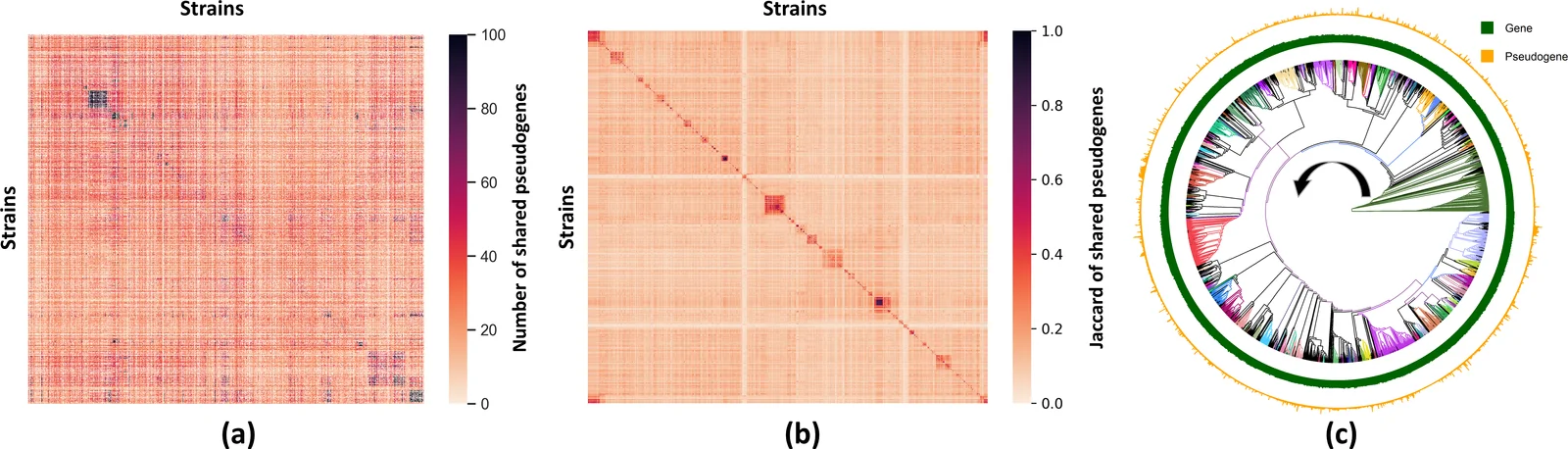
Strain pairwise analysis of shared pseudogenes. Heatmaps show (a) absolute count and (b) Jaccard index of the number of shared pseudogenes between pairs of strains. The mean and maximum values are as follows: mean = 33, maximum = 933 and mean = 0.11, maximum = 1 in (a) and (b), respectively. (c) Phylogenetic tree of *P. aeruginosa* strains: the green and orange layers on top of the tree indicate the number of genes and pseudogenes, respectively, for the strains at the tips of the tree. The branches are colored based on MLST as follows. Each sequence type (ST) was transformed into a random hex number which is translated into a color. STs that were assigned to less than five different strains were filtered out. Altogether there are 135 colors in the tree. Black color is assigned to strains that have no ST information or belong to STs that were filtered out. The order of the strains in rows (up to bottom) and columns (left to right) of both heatmaps is identical and corresponds to the order of the tips (indicated with an arrow) of the phylogenetic tree shown in (c).

We calculated the number of shared pseudogenes between all pairs of strains and plotted the results as heatmaps of absolute counts (Fig. 5a) and of Jaccard index values (Fig. 5b). The order of the strains in rows and columns of the heatmaps corresponds to the order of the tips of the phylogenetic tree shown in Fig. 5c. Most of the strain pairs share few, if any, pseudogenes, as evidenced by both heatmaps. Interestingly, higher values of Jaccard index are concentrated along the diagonal of the graph, indicating that some phylogenetically close strains share a significantly higher number of pseudogenes than random pairs in the data set.

### Neighborhood analysis of pseudogenes and genes inside mixed clusters maps patterns of their phylogenetic relationships

Finally, we analyzed the phylogenetic neighborhood of orthologous genes and pseudogenes within genes-pseudogenes mixed clusters. As before, we pulled the clusters from which the representative sequences originated. We kept 2,653 mixed clusters whose original sizes, for both genes and pseudogenes, ranged between 3 and 2,500. We then analyzed each mixed cluster as follows. First, we overlayed the genes and pseudogenes in the cluster to the tips of the tree (Fig. S4) that correspond to the strains they belong to. Second, we traversed the tree in the linear order of the tips to allocate tips containing gene/pseudogene. Then, we examined their neighborhood (five strains forward and backward) and labeled each tip by one of the labels with the following priority: (1) with same type—there is another tip containing gene/pseudogene in the neighborhood; (2) with opposite type—there is another tip containing pseudogene/gene in the neighborhood; or (3) alone—there are no tips with either gene or pseudogene in the neighborhood. Finally, we computed the proportions of each label, separately for tips containing genes and tips containing pseudogenes.

We then examined the association between label proportion and cluster size. We found that as cluster size grows, the proportion of the labels “alone” and “with opposite type” drops, while for label “with same type” it increases, for both genes (Fig. S6a) and pseudogenes (Fig. S6b). We did not observe any correlation between the proportion of the labels for genes versus pseudogenes within clusters (Fig. S7).

**Fig 6:**
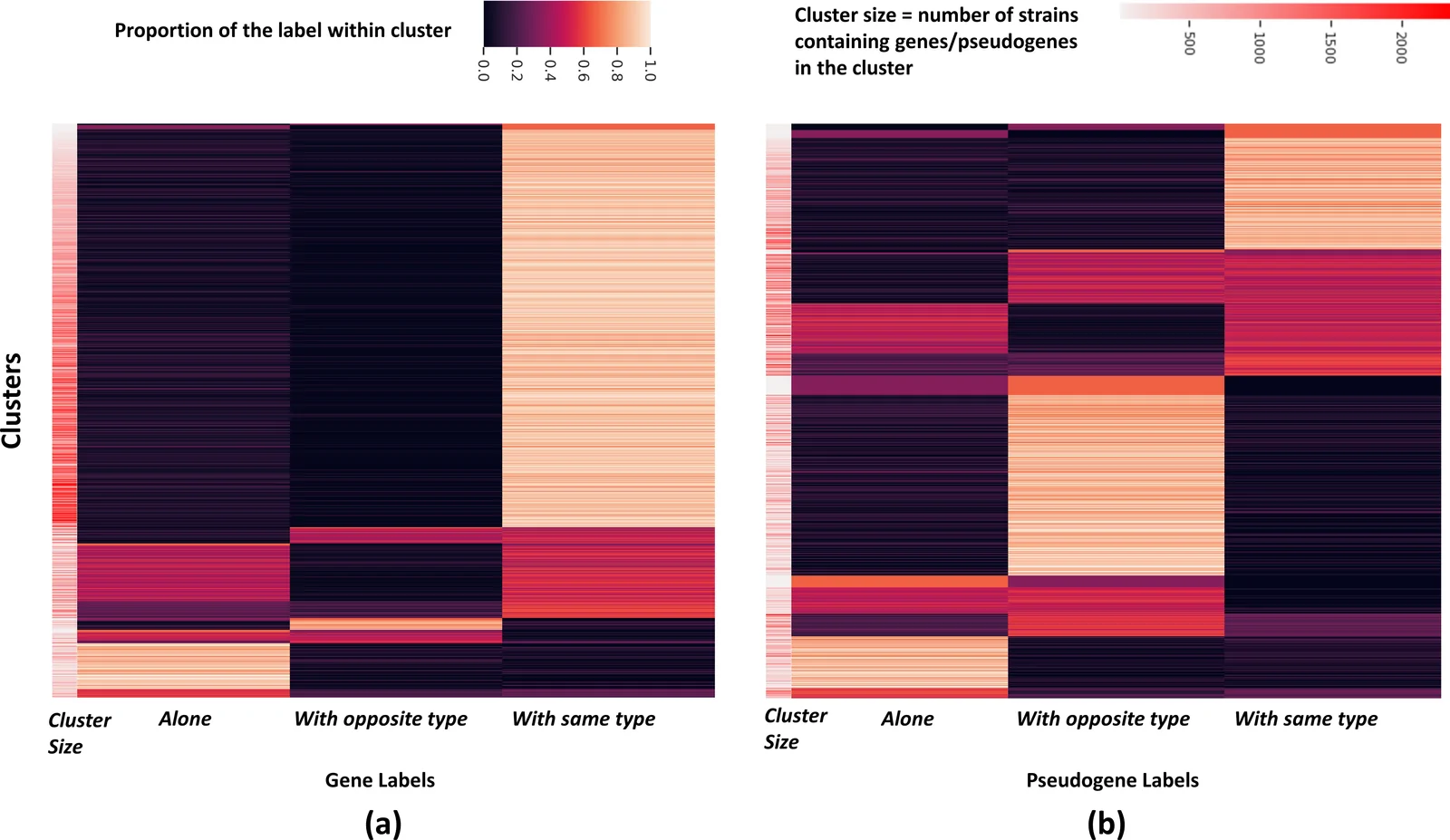
Distribution of genes and pseudogenes neighborhood labels within clusters visualized through heatmaps. These heatmaps represent 2,653 gene-pseudogene mixed clusters in the rows and three possible labels in the columns for (a) genes or (b) pseudogenes. The color of each cell indicates the proportion of the label (column) within the cluster (row). The left bars in each heatmap represent cluster size, i.e., the number of strains containing genes/pseudogenes in the cluster. The clusters were sorted by the proportions of different labels from high to low in the following priority: with same type*,* with opposite type, and alone.

Lastly, Fig. 6 provides additional visualization of neighborhood label distribution for genes and pseudogenes within clusters. A total of 70.3% of the clusters have a high ratio of genes labeled “with same type” (Fig. 6a), and these clusters are large in size (size corresponds to the number of unique strains represented by the sequences in the cluster). The number of clusters that have a high proportion of “alone” pseudogenes or genes is similar (bottom of the heatmaps in Fig. 6a and b); however, for pseudogenes, 56% of the clusters have medium-high proportions of pseudogenes labeled as “with opposite type” (Fig. 6b), indicating the strains containing these pseudogenes are adjacent to strains that contain their orthologous genes but not pseudogenes.

## DISCUSSION

Identification and accurate annotation of pseudogenes are vital for comparative genomics studies and can lead to meaningful insights and discoveries about evolutionary events, horizontal transfer events, and regulatory mechanisms. The task for accurate annotation remains a major challenge due to, for example, differences between genomic databases, inconsistent definition of pseudogenes, and sequencing errors (9).

The goal of the current study was to explore the characteristics and phylogenetic patterns of already published annotated pseudogenes in the bacterium *P. aeruginosa* and interpret their reliability rather than identifying new pseudogenes. Previous works have shown that pseudogenes are far more scarce in free-living prokaryotic genomes than genes and tend to form between 1% and 5% of the genome (3, 16). In our collection, the number of pseudogenes varied widely, from 0 to 1,970 pseudogenes per strain, such that pseudogenes constitute about 26% of total genes and pseudogene content in some strains. Such high numbers may indicate real biological signals. However, it is also possible that many of the annotated pseudogenes result from assembly artifacts or annotation errors.

In our analysis, we found that pseudogene counts are higher in strains with low assembly levels than in strains with high assembly levels, as the first group of genomes probably contains more broken genes, which may lead to misannotation of genes as pseudogenes. Similarly, higher pseudogene counts were observed for strains in clinical versus environmental isolation type. Of note, in both comparisons, the groups with higher pseudogene counts (i.e., low assembly level and clinical isolation type) were larger by far than those with lower pseudogene counts (i.e., high assembly level and environmental isolation). Thus it will be interesting to see if a similar phenomenon repeats in other bacterial species with larger collections of strains with relevant metadata. Furthermore, as the current state is that most of the strains are of low quality, we rely on the assumption that “errors” in annotation are spread along these poorly assembled genomes, and when taken together they can be used to identify the noise.

Clustering of the gene and the pseudogene sequences provided more insights about orthology within each group. Specifically, the analysis of sequence length variation within gene and pseudogene clusters showed much higher variation in the latter group, which could imply, for example, that pseudogenes are formed by various mechanisms that affect their length, that this is an artifact of the pseudogene annotation pipelines in the wrong identification of bona-fide pseudogenes, or misannotation of broken bona-fide genes as pseudogenes.

To study the orthology relationships of genes and pseudogenes, we performed an additional round of clustering using the representative sequences of gene and pseudogene clusters. Notably, clustering all genes and pseudogenes (30 million sequences) on the DNA level is computationally intensive. Using representatives of orthologous sequences instead made this task feasible. This clustering analysis identified three types of clusters, gene-only, pseudogene-only, and a mix of both genes and pseudogenes. Surprisingly, there were 20,419 pseudogene-only clusters, representing pseudogenes that are not homologous to any of *P. aeruginosa* genes. In this analysis, we used CD-HIT-EST with 80% similarity threshold due to computational constraints. As pseudogenes tend to accumulate nucleotide changes, it is possible that such a high threshold makes it difficult to detect the phylogenetic relationship of highly mutated pseudogenes with the parental gene. Future directions could be examining other clustering algorithms or thresholds or trying to identify their origin in other bacteria or bacterial phages.

Interestingly, for mixed clusters, we observed various combinations of gene and pseudogene clusters’ sizes. For example, there are clusters with a high number of genes and a low number of pseudogenes, or vice versa; there are also clusters with high numbers of both genes and pseudogenes. These different patterns could indicate different evolutionary events that different genes undergo, for example, higher rates of horizontal transfer events, as it was shown that a large fraction of prokaryote pseudogenes arose from failed horizontal transfer events (16).

Focusing on pseudogene-only and mixed clusters, we classified each pseudogene sequence based on the type and size of the cluster it belongs to (largely corresponds to the number of its orthologous pseudogenes and genes). The proportion of pseudogenes that have both orthologous pseudogenes and genes was the highest for most strains, followed by relatively high proportions of pseudogenes with only orthologous pseudogenes. Nevertheless, we also identified a set of strains for which the proportion of the complement situation, i.e., pseudogenes without any orthologous pseudogenes (with or without orthologous genes), is very high. For example, the strain *JMM* has 234 pseudogenes, where 113 (48%) of them are singletons with no orthologous pseudogenes or genes. For the strain *E* 6_*London*_17_*V I M* _2_12_12, 199 of its pseudogenes (30%) have orthologous genes but no orthologous pseudogenes. For 42 strains, such proportions exceed 20%. Such high proportions could imply poor assembly quality and high annotation error of pseudogenes. This small group of strains has 237.7 scaffolds on average, while the average across all strains is 181.4; a high number of scaffolds in the genome assembly is a potential indication for assembly errors (21), which has a direct effect on gene annotation.

To study in depth the phylogenetic patterns of pseudogenes across the *P. aeruginosa* strains, we generated a phylogenetic tree of the strains. We then analyzed the homogeneity of the number of pseudogenes between phylogenetically related strains by traversing the tree tips, relying on the assumption that the order of the tips provides a good approximation to phylogenetic closeness between the strains. For comparison, we also examined the grouping of strains by MLST, BioProject, and random. We found a significant statistical difference between random grouping to grouping by tree, MLST, and BioProject, supporting the hypothesis that phylogenetically related strains show higher homogeneity in the number of pseudogenes compared to random groups of strains. As this analysis looked only at the numbers of pseudogenes but not their identity, we next analyzed the number of shared pseudogenes for all strain pairs. Examination of the distribution of absolute counts and Jaccard indexes of shared pseudogenes revealed that phylogenetically close strains share significantly more pseudogenes than the average of all pairs; however, this was not evident in all regions of the phylogenetic tree.

Lastly, we aimed to study the phylogenetic neighborhood of pseudogenes within genes-pseudogenes mixed clusters. We found that 31% of the clusters have medium-high proportions of pseudogenes labeled as “alone” (Fig. S8 and S9). Such scattering of pseudogenes across the tree could indicate failed horizontal transfer events (16). Fifty-six percent of clusters have medium-high proportions of pseudogenes that are adjacent to their orthologous genes but no pseudogenes (i.e., with opposite type), which raises speculation that these pseudogenes are, in fact, genes that were misannotated (Fig. S10). Twenty-two percent of clusters have high proportions of pseudogenes labeled as “with same type” (Fig. S11 and S12). These pseudogenes could be examples of evolutionary preserved pseudogenes that have acquired a functional role. However, we cannot rule out the possibility that these are disappearing pseudogenes that have not had sufficient evolutionary time.

In summary, in this study, we comprehensively explored different characteristics of annotated pseudogenes in *P. aeruginosa* strains and compared them to those observed in genes. Utilizing clustering and phylogenetic methods, we have pointed to possible annotation errors of pseudogenes. Thus similar methodologies could be integrated into annotation pipelines to reduce such errors. Future studies could be expanded to other bacterial and non-bacterial species, with rich collections of strains, for examining and comparing pseudogene patterns. Another interesting research direction is to examine the sequence, structure, genomic context, and function of the annotated pseudogenes that fall under different categories, by relying on, for example, the function of orthologous genes. Examination of sequence can reveal the reason for annotation as pseudogenes; for example, fragmented sequences on the end of contigs or point mutations that inserted a stop codon in the sequence. Examination of structure can indicate the completeness of protein domains, while the context can imply if they are part of mobile elements. Examination of function can reveal which gene families are more prone to pseudogene generation (16). Such in-depth study of the structural and functional features of the annotated pseudogenes can provide insights that can be further incorporated into pseudogene annotation pipelines.

## MATERIALS AND METHODS

### Data collection

We downloaded the 4,699 *P*. *aeruginosa* strains list from the RefSeq database on the NCBI website (22) (on July 2019). We retrieved the FTP address for each strain from the summary file and used it to download annotation files that include (1) nucleotide sequences of coding genes in Fasta format (cds_from_genomic.fna), (2) protein sequences in Fasta format (protein.faa), and (3) a table that contains locations and attributes for genomic features, e.g., coding genes and pseudogenes (feature_table.txt). In addition, for each strain, we retrieved information about the genome-assembly level from the NCBI genome browser and isolation type from the NCBI isolates browser.

### Pre-processing of the data

For each strain, we generated two files combining the data from the above-mentioned files, genes.csv and pseudo_genes.csv. To create the genes.csv file, we extracted from the feature_table.txt file records which are labeled as (1) "gene" and "protein_coding" or (2)"CDS" and "with_protein" (in columns "feature" and "class", respectively). Using the "locus_tag" label, we merged the records of genes and CDSs. For each combined record, we added a DNA sequence from cds_from_genomic.fna and a protein sequence from protein.faa. To create the pseudo_genes.csv file, we extracted from the feature_table.txt file records which are labeled as (1) "gene" and "pseudogene" or (2)"CDS" and "without_protein" (in columns "feature" and "class", respectively). Using the "locus_tag" label, we merged the records of genes and CDSs. We added a DNA sequence for each combined record from cds_from_genomic.fna.

### Clustering of coding genes and pseudogenes

We collected the protein sequences of coding genes across all strains into a single FASTA file containing 28,948,105 sequences. We defined the header convention for each sequence to reference it back to its strain more easily. We added indices to the header in the following way: i|j , where *i* is the index of the strain in the context of all strains and *j* is the index of the sequence in the context of all sequences that belong to the same strain. An index file was generated to map indices to strains. The order of the sequences in the table of features defined the indices of the sequences within a strain. We clustered the sequences in this file using CD-HIT (17) with the following parameters: similarity threshold of 70%, word size of 5, and the slow mode version, which is considered a more accurate version of the algorithm. Similarly, we collected the DNA sequences of pseudogenes across all strains into a single FASTA file containing 1,113,358 sequences. We applied CD-HIT-EST, suitable for nucleotide sequences, to cluster the sequences in the file with the following parameters: similarity threshold of 80%, word size of 5, and the slow mode version. The genes and pseudogenes FASTA files, as well as the files containing the clustering results, can be found at https://zenodo.org/record/8202450.

### Mapping pseudogenes to genes (clustering of representatives)

To map each pseudogene to its ortholog gene, we first extracted the DNA sequences of the representatives of the gene and pseudogene clusters mentioned above into a single FASTA file of 86,273 sequences, such that the header of each sequence contains the following information:


Gene∖Pseudogene|Clusterindex|Clustersize|Sequencelength|Strainindex|Sequenceindex

We then applied CD-HIT-EST to cluster the sequences with the following parameters: similarity threshold of 80%, word size of 5, and the slow mode version. When analyzing these clusters, we used the information stored in the header of each representative sequence to retrieve the associated sequences (i.e., the respective members from the gene/pseudogene clusters).

### Generation of phylogenetic tree

We parsed gene clustering results to identify core genes, i.e., clusters in which more than 90% of the strains are present in one copy (4,651 core clusters). For each core cluster, we collected the DNA sequences of the genes into a single FASTA file. We then aligned these sequences using the MAFFT program (version 7.471) with default parameters (23) and applied Gblocks (version 0.91b) (24) to remove poorly aligned positions (with parameters −*t* = *d,* − *b*5 = *a*). Using an in-house code, we removed all the invariant positions (excluding gaps) from the alignment and padded the alignment with gaps for strains in which the core gene was missing. Finally, we concatenated all the alignments into a single alignment of 1,369,462 nucleotides. We applied the program FastTree (25) on the core gene alignment file using the -gtr option to generate the phylogenetic tree of all the strains in the analysis. The generated tree was written into a Newick file, in which each strain is represented by a leaf and marked with its assigned index.

### Visualization of the phylogenetic tree

We visualized and annotated the phylogenetic tree using the R packages ggtree (26) and its extension ggtreeExtra (27). We incorporated MLST (19) into the tree view to highlight the relationships between different strains. We collected the MLST data from the Pseudomonas genome database (28) and grouped the strains by the sequence type (ST) number. STs that were assigned to less than five different strains were filtered out. Each ST was transformed into a random hex number which was translated to a color. In addition to MLST data, we have grouped the strains by their respective BioProject. The BioProject data were collected from the NCBI genome browser. Similarly to the MLST, BioProjects that were assigned to less than five different strains were filtered out.

### Computing pseudogenes shared among strains

Let *C_i* be the group of pseudogene clusters that contain sequence/s from strain *i*. For each pair of strains (*i*,*j*) we calculated (1) the number of their shared pseudogenes as *|C⁢_⁢i∩C⁢_⁢j|* and (2) Jaccard index (29):


$$Jaccard(i,j)=\frac{|C_{i}\cap C_{j}|}{|C_{i}|+|C_{j}|-|C_{i}\cap C_{j}|}.$$

The results are organized into a matrix in which rows and columns correspond to strains, ordered as their matching tips in the phylogenetic tree.

### Neighborhood analysis of pseudogenes and genes inside mixed representative clusters

To further investigate the phylogenetic neighborhood of genes and pseudogenes, we have analyzed mixed clusters of pseudogenes and genes generated by clustering of representatives, as explained above. In this analysis, we omitted outlier clusters whose original sizes (i.e., unique strains represented by the sequences in the cluster) were less than 3 or more than 2,500. For each of the remaining clusters, we performed the following: we overlaid the genes and pseudogenes in the cluster to the tree’s tips representing the strains they belong to. Then while traversing the tree in the linear order of the tips (i.e., strains), we stopped at each tip containing either a gene, a pseudogene, or both, and examined its neighborhood consisting of five strains forward and five strains backward. We then labeled each tip containing gene/pseudogene with one of the following labels, prioritized from 1 to 3: (1) with same type—there is another tip containing gene/pseudogene in the neighborhood; (2) with opposite type—there is another tip containing pseudogene/gene in the neighborhood; and (3) alone—there are no tips with either gene or pseudogene in the neighborhood. We then computed the proportions of each label separately for tips containing genes and tips containing pseudogenes.

### Software and packages

The statistical analysis was performed using specific python packages: (1) SciPy for the Kruskal-Wallis test, Wilcoxon-Mann-Whitney test and coeﬃcient of variation, and (2) scikit-posthocs for Dunn’s test (30).

## SUPPLEMENTAL MATERIAL

Figures S1 to S12, Tables S2 to S4 spectrum.01704-23-s0001.pdf Supplemental figures and tables.

Table S1 spectrum.01704-23-s0002.xlsx List of strains included in this study.

ASM does not own the copyrights to Supplemental Material that may be linked to, or accessed through, an article. The authors have granted ASM a non-exclusive, world-wide license to publish the Supplemental Material files. Please contact the corresponding author directly for reuse.
