# Supplementary material for: A large-scale phylogeny-guided analysis of pseudogenes in Pseudomonas aeruginosa bacterium
Source: Microbiol Spectr. 2023 Sep 26;11(5):e01704-23. doi: 10.1128/spectrum.01704-23 (PMC10580986; doi:10.1128/spectrum.01704-23)
Supplement: Figures S1 to S12, Tables S2 to S4 — Supplemental figures and tables. [file spectrum.01704-23-s0001.pdf]

A large-scale phylogeny-guided analysis of pseudogenes in *Pseudomonas aeruginosa* bacterium

Supplementary material

Nimrod Cohen and Isana Veksler-Lublinsky

1 Supplementary figures

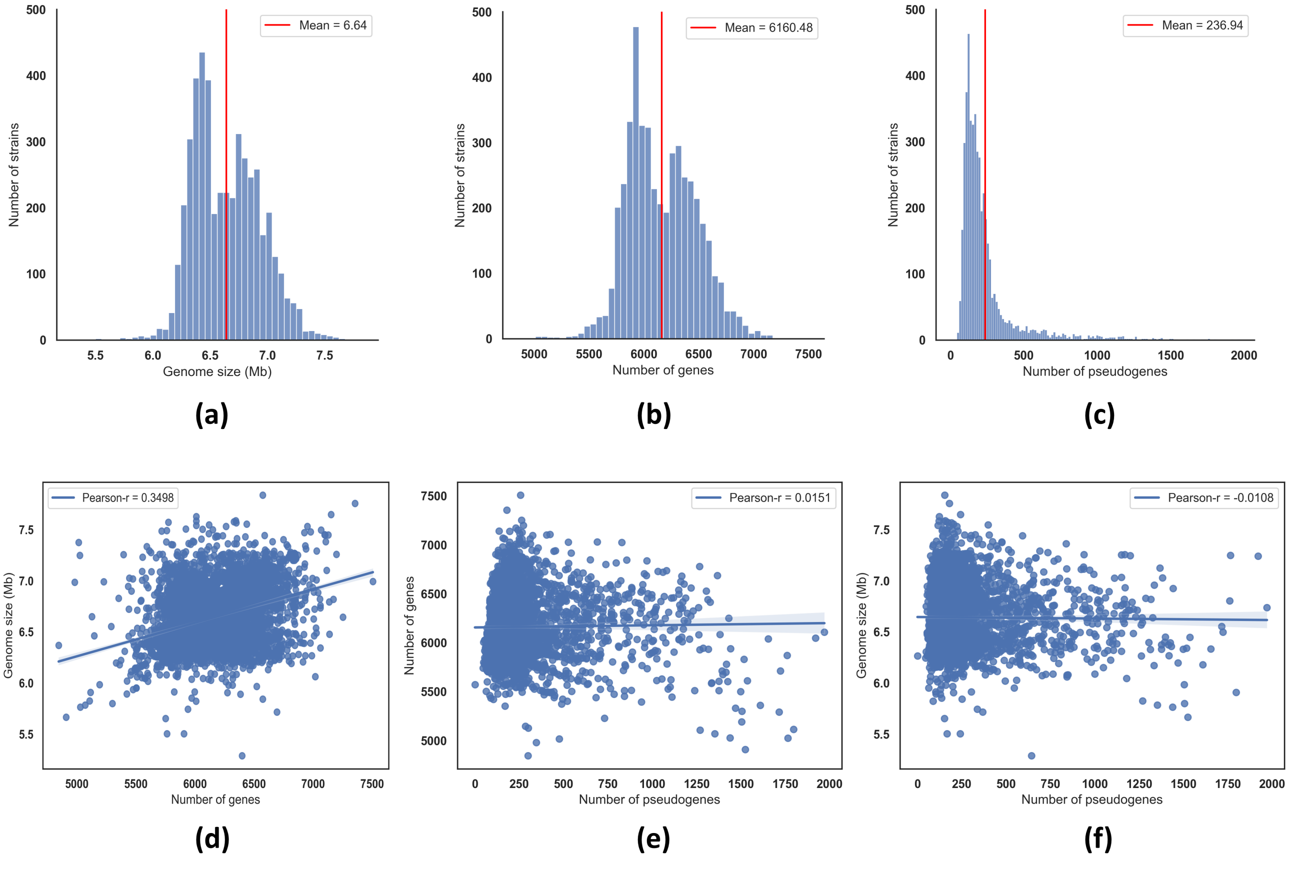

Fig. S1: **Characteristics of *P.aeruginosa* strains.** Distributions of (a) genome size, (b) number of genes, and (c) number of pseudogenes across 4699 *P.aeruginosa* strain. (d-f) Correlations between genome size, number of genes, and number of pseudogenes. (d) Number of genes (x-axis) versus genome size (y-axis); (e) number of pseudogenes (x-axis) versus number of genes (y-axis); and (f) number of pseudogenes (x-axis) versus genome size (y-axis). Each dot in (d-f) corresponds to a strain in the dataset.

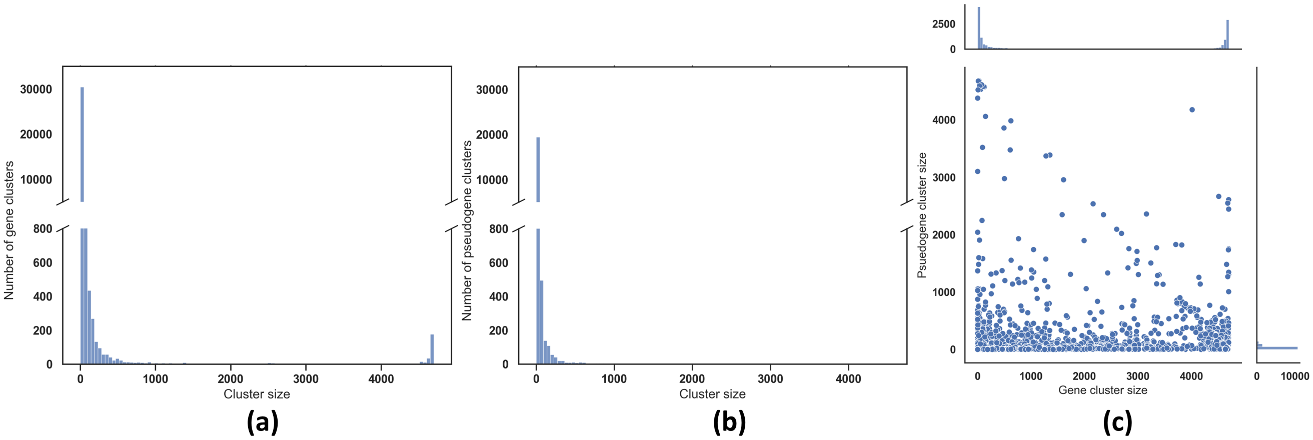

Fig. S2: **Examining cluster size from which the representative sequences originated.** Size corresponds to the number of unique strains represented in the cluster. Size distribution of (a) gene-only clusters and (b) pseudogene-only clusters; size corresponds to the number of unique strains represented in the cluster. (c) Mixed clusters, with two representatives, one from each type. Each dot represents a cluster. Sizes of the clusters of origin of the gene representative and the pseudogene representative are indicated on the x-axis and y-axis, respectively. The plots at the margins show the size distribution of gene and pseudogene clusters (equivalent to (a) and (b), respectively). Both distributions are similar to the distributions of all gene and pseudogene clusters (Figures 3(a) and 3(b), respectively).

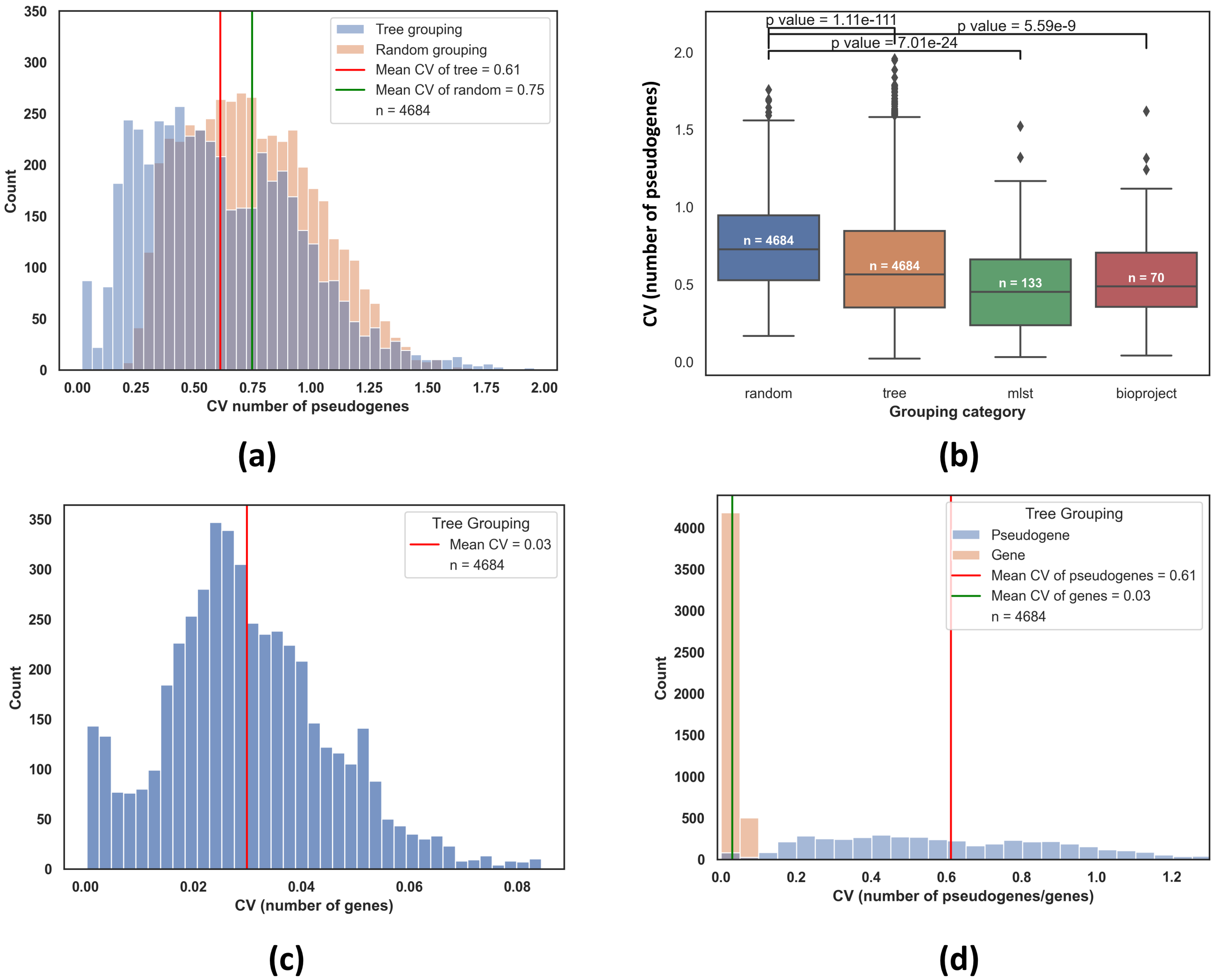

Fig. S3: **Distribution of coefficient of variation (CV) of the number of pseudogenes or genes for different groupings of strains.** (a) CV of number of pseudoegenes; grouping by phylogenetic tree versus random grouping. (b) Comparison of CV distribution between different groupings (p-values are computed with Mann-Whitney U rank test). The box and whiskers span interquartile range (IQR) and 1.5 $\times$ IQR, respectively, and diamonds represent outliers. (c) CV of number of genes; grouping by phylogenetic tree. (d) CV of the number of genes versus CV of number of pseudogenes; grouping by phylogenetic tree. Group size is 15. Complementary to Figure 6.

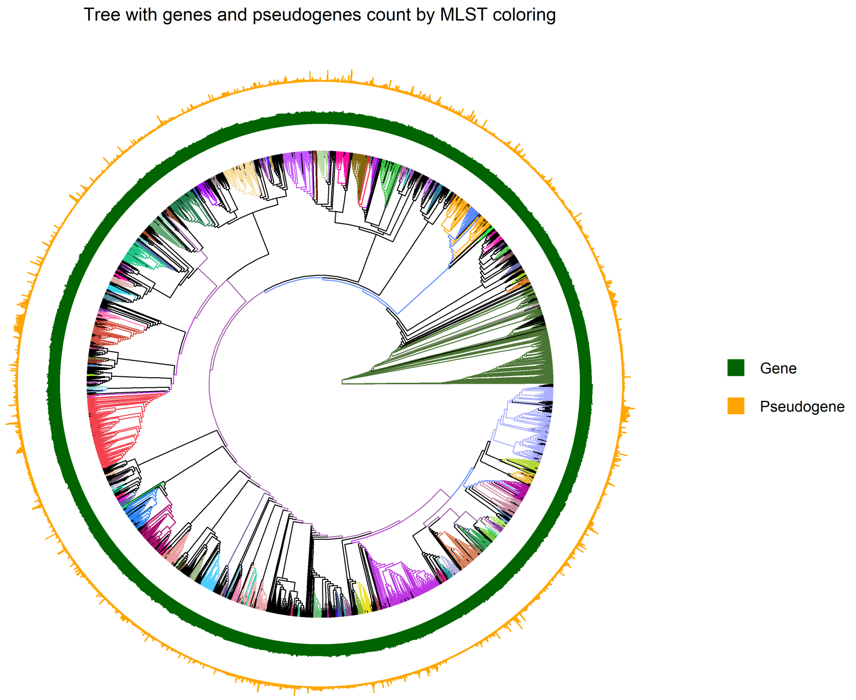

Fig. S4: **Phylogenetic tree of *P. aeruginosa* strains, colored by MLST, showing genes and pseudogenes counts across strains.** The green and orange layers on top of the tree indicate the number of genes and pseudogenes, respectively, for the strains at the tips of the tree. The branches are colored based on MLST as follows. Each ST was transformed into a random hex number which is translated into a color. STs that were assigned to less than five different strains were filtered out. Altogether there are 135 colors in the tree. Black color is assigned to strains that have no ST information or belong to STs that were filtered out.

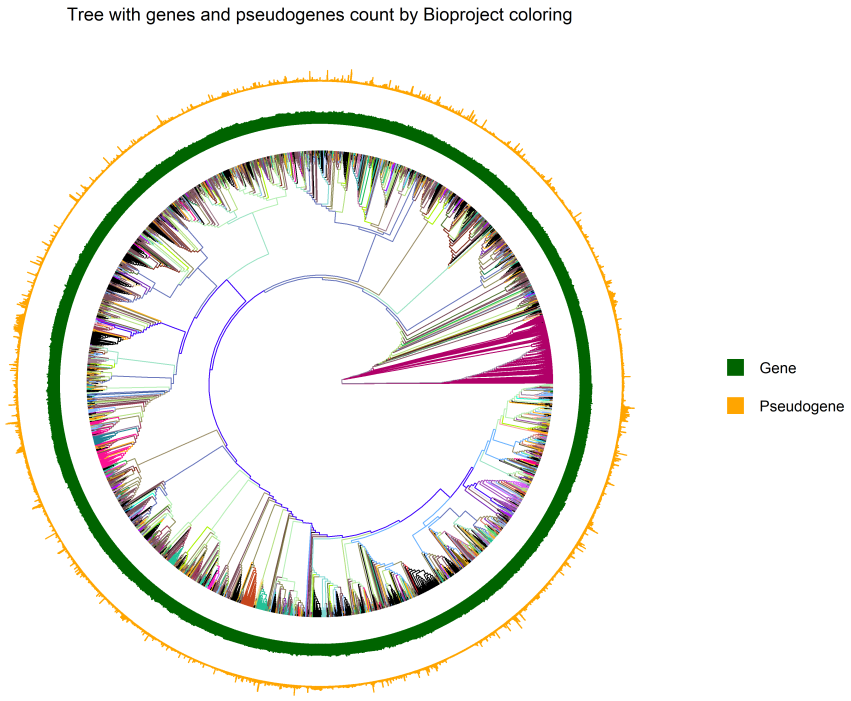

Fig. S5: **Phylogenetic tree of *P. aeruginosa* strains, colored by BioProject, showing genes and pseudogenes counts across strains.** The green and orange layers on top of the tree indicate the number of genes and pseudogenes, respectively, for the strains at the tips of the tree. The branches are colored based on BioProject as follows. Each Bioproject was transformed into a random hex number which is translated into a color. Bioprojects that contain less than five different strains were filtered out. Altogether there are 71 colors in the tree. Black color is assigned to strains that belong to Bioprojects that were filtered out.

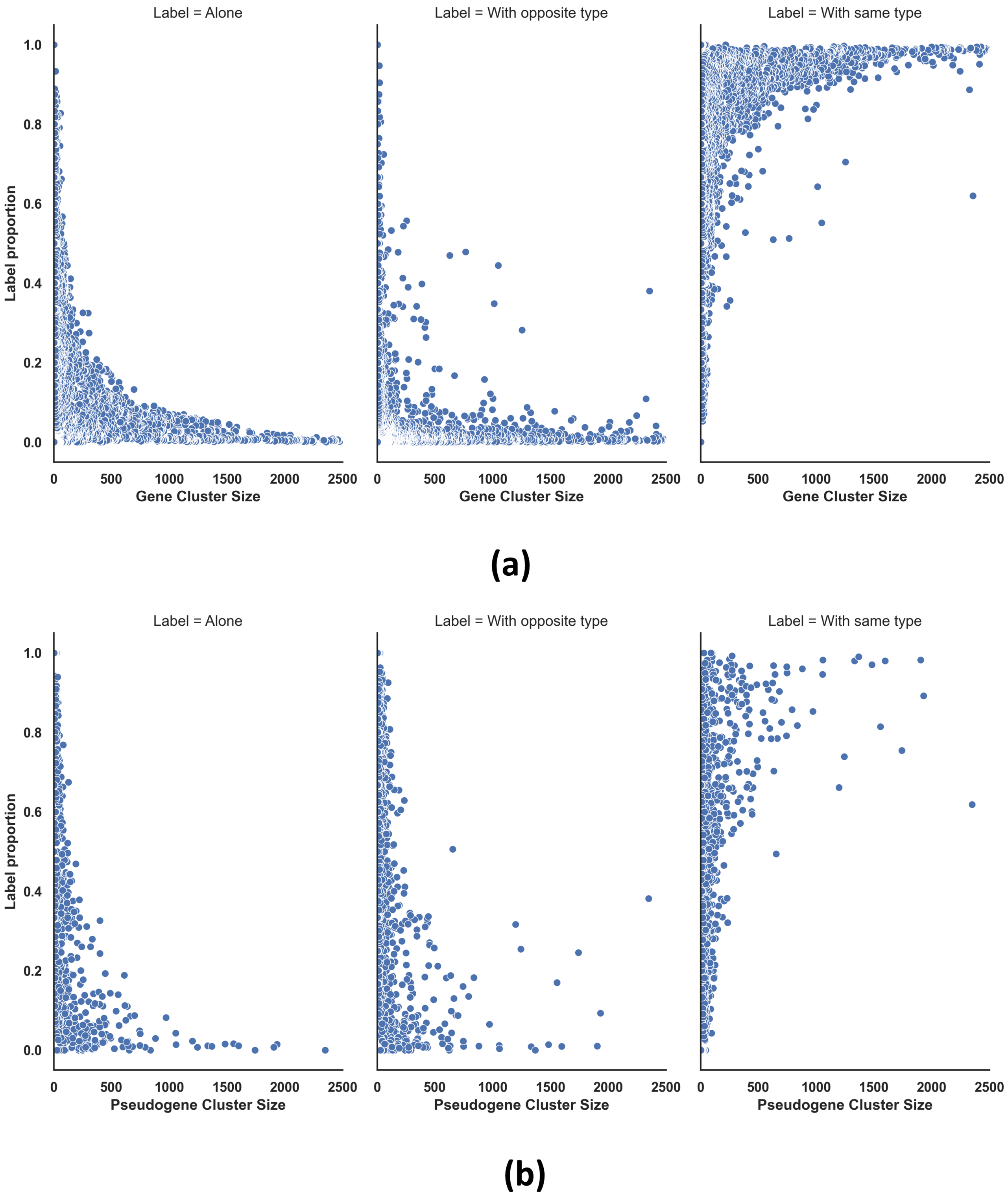

Fig. S6: **Association between the proportion of neighbourhood-labels to the size of gene/pseudogene clusters.** Scatter plots show on the x-axis cluster size for (a) genes and (b) pseudogenes, versus the proportions of the indicated labels on the y-axis. Each dot represents one cluster out of 2,653 gene-pseudogene mixed representative clusters, for which the original cluster sizes (i.e., number of strains) of both genes and pseudogenes ranged between 3-2500. The labels *alone*, *with same type* and *with opposite type* are indicated on top of each plot. For each cluster, the sum of the proportions of the three labels sums up to 1, separately for genes and for pseudogenes.

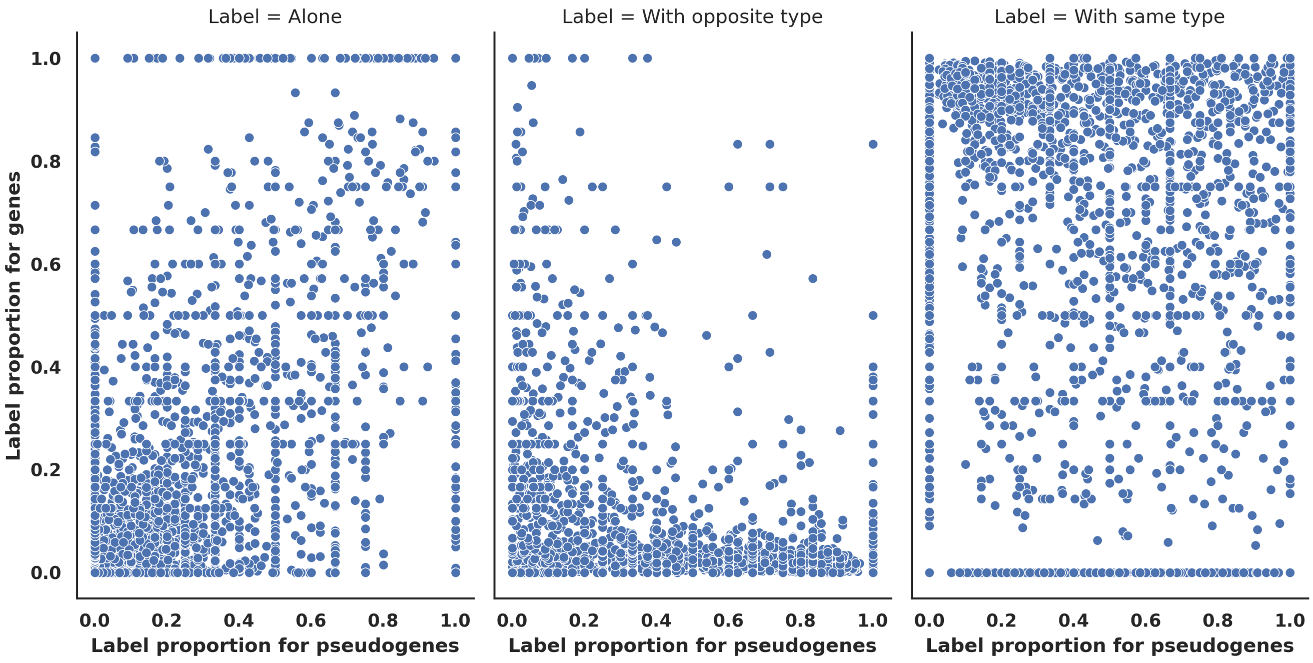

Fig. S7: **Association between neighborhood-label proportions for genes versus pseudogenes.** Scatter plots show the label proportion of pseudogenes (x-axis) versus the label proportion of genes (y-axis) for the indicated labels *alone*, *with same type*, and *with opposite type*. Each dot represents one cluster out of 2,653 gene-pseudogene representative clusters, for which the original cluster sizes (i.e., number of strains) of both genes and pseudogenes range between 3-2500.

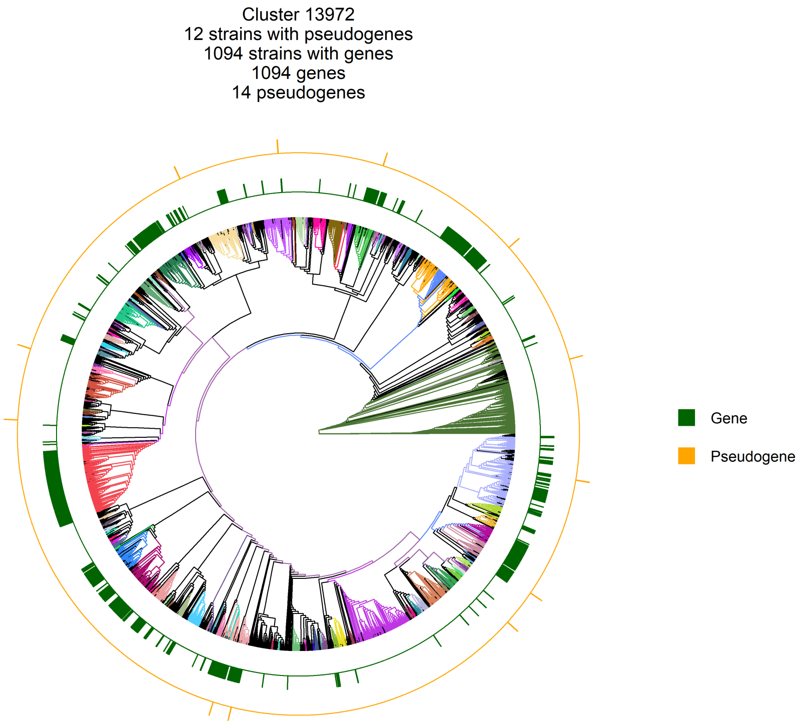

Fig. S8: **Phylogenetic tree of *P. aeruginosa* strains with gene/pseudogene information for a cluster with a high proportion of pseudogenes labeled as *alone*.** The branches of the tree are colored by MLST groups as in Figure S4. The green and orange layers on top of the tree indicate the existence of genes and pseudogenes, respectively, in the strains at the tips of the tree.

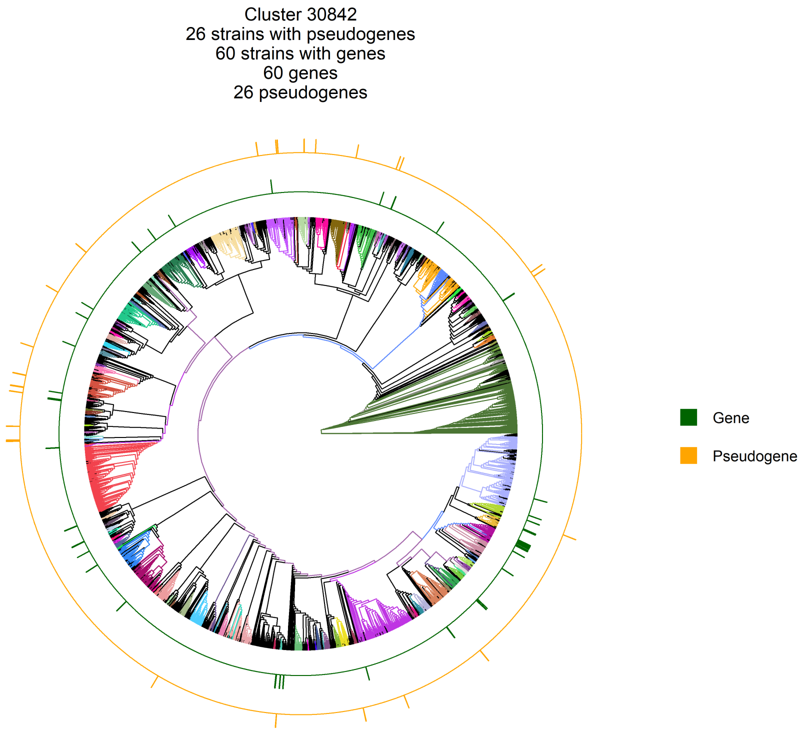

Fig. S9: **Phylogenetic tree of *P. aeruginosa* strains with gene/pseudogene information for a cluster with a high proportion of pseudogenes labeled as *alone*.** The branches of the tree are colored by MLST groups as in Figure S4. The green and orange layers on top of the tree indicate the existence of genes and pseudogenes, respectively, in the strains at the tips of the tree.

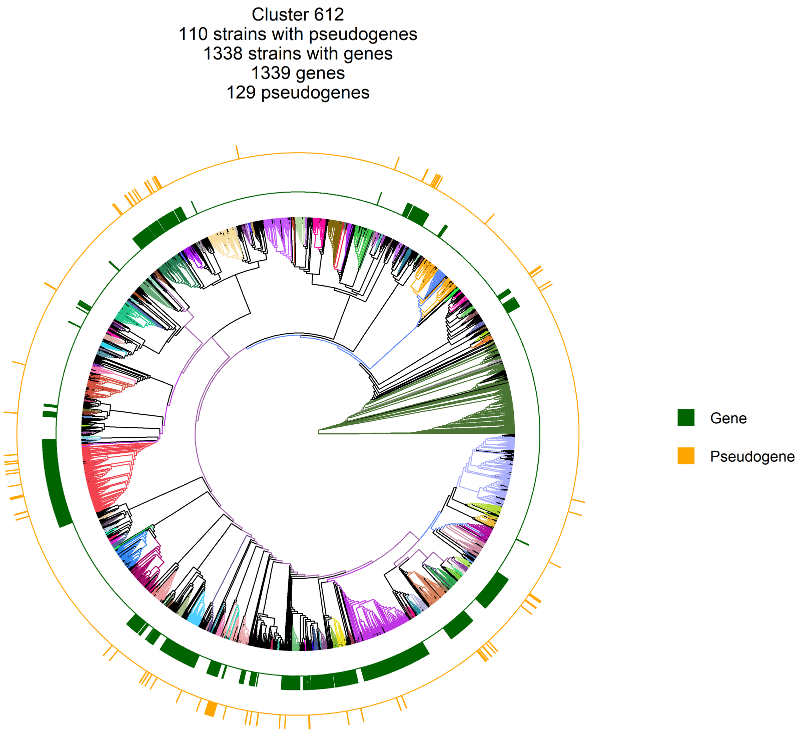

Fig. S10: **Phylogenetic tree of *P. aeruginosa* strains with gene/pseudogene information for a cluster with a medium-high proportion of pseudogenes labeled as *with-opposite-type*.** The branches of the tree are colored by MLST groups as in Figure S4. The green and orange layers on top of the tree indicate the existence of genes and pseudogenes, respectively, in the strains at the tips of the tree.

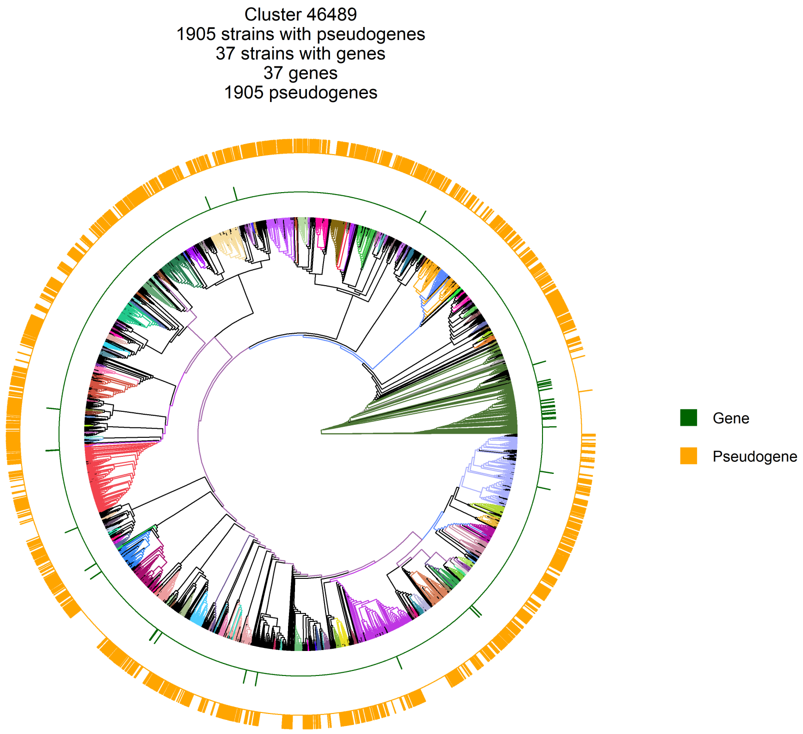

Fig. S11: **Phylogenetic tree of *P. aeruginosa* strains with gene/pseudogene information for a cluster with a high proportion of pseudogenes labeled as *with-same-type*.**The branches of the tree are colored by MLST groups as in Figure S4. The green and orange layers on top of the tree indicate the existence of genes and pseudogenes, respectively, in the strains at the tips of the tree.

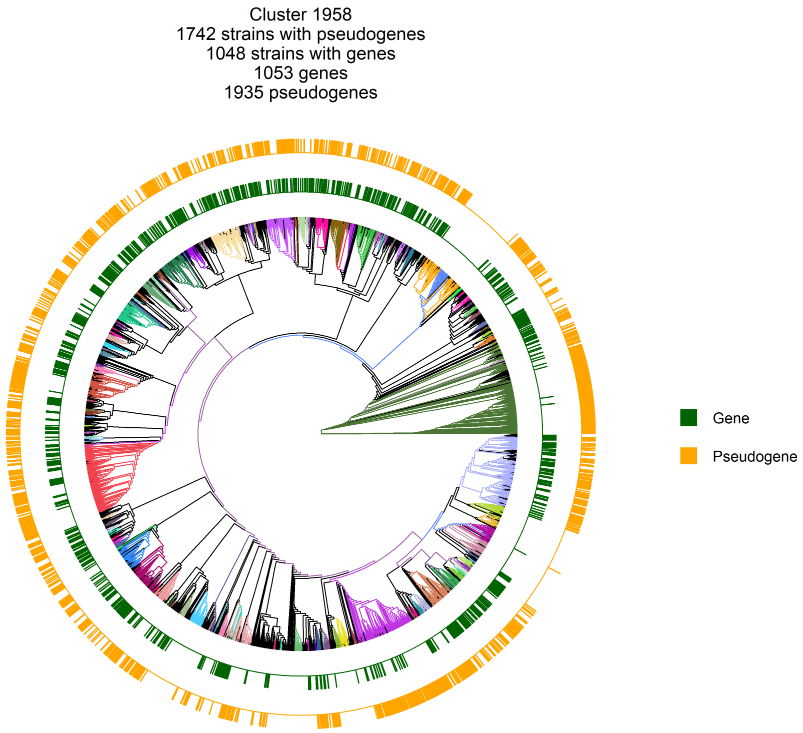

Fig. S12: **Phylogenetic tree of *P. aeruginosa* strains with gene/pseudogene information for a cluster with a high proportion of pseudogenes labeled as *with-same-type*.** The branches of the tree are colored by MLST groups as in Figure S4. The green and orange layers on top of the tree indicate the existence of genes and pseudogenes, respectively, in the strains at the tips of the tree.

2 Supplementary tables

Table S2: **Summary of clustering results**

	Genes clustering	Pseudogenes clustering	Representatives clustering
Number of sequences	28,948,105	1,113,358	86273
Number of clusters	48968	37305	67349
Core clusters (>90% of strains)	5011	11	
Accessory	43957	37294	
Singletons (Only one strain)	13021	17555	
Singletons (Only one member)	12916	17374	

Table S3: **Distribution of clusters based on the number of representatives and their origin.** Cluster type corresponds to the origin of the representatives: gene-only, pseudogene-only, or a mix of both.

	Number of representatives	Cluster type	Clusters count	% from total clusters
0	1	gene	32931	48.896
1	1	pseudo	20419	30.318
2	2	gene	438	0.650
3	2	mixed	10036	14.901
4	3	gene	39	0.058
5	3	mixed	2590	3.846
6	4	gene	9	0.013
7	4	mixed	582	0.864
8	5	gene	3	0.004
9	5	mixed	202	0.300
10	6	mixed	55	0.082
11	7	mixed	22	0.033
12	8	mixed	9	0.013
13	9	mixed	7	0.010
14	10	mixed	2	0.003
15	11	mixed	2	0.003
16	12	mixed	1	0.001
17	13	mixed	1	0.001
18	14	mixed	1	0.001

Table S4: **Distribution parameters related to Figure 5(c).**

	Pseudogene-only-single	Mixed-single	Pseudogene-only-multiple	Mixed-multiple
count	4699	4699	4699	4699
mean	0.008	0.003	0.241	0.748
std	0.04	0.01	0.055	0.067
min	0	0	0	0
25%	0	0	0.208	0.713
50%	0	0	0.245	0.748
75%	0.004	0.003	0.278	0.785
max	0.8	0.304	0.471	0.921
